# Supplementary material for: Synthesis and Characterization of Magnetic Drug Carriers Modified with Tb3+ Ions
Source: Nanomaterials (Basel). 2022 Feb 26;12(5):795. doi: 10.3390/nano12050795 (PMC8912105; doi:10.3390/nano12050795)
Supplement: Supplementary file 1 [file nanomaterials-12-00795-s001.zip › nanomaterials-1553571-supplementary.pdf]

Supporting Information

Synthesis and Characterization of Magnetic Drug Carriers Modified with Tb^{3+} Ions

Dorota Nieciecka *, Aleksandra Rękorajska, Dariusz Cichy, Paulina Końska, Michał Żuk and Paweł Krysiński

Faculty of Chemistry, University of Warsaw, Pasteura 1, 02-093 Warsaw, Poland; olumbalum@gmail.com (A.R.); dariusz.cichy@student.uw.edu.pl (D.C.); p.konska@student.uw.edu.pl (P.K.); mt_zuk@chem.uw.edu.pl (M.Z.); pakrys@chem.uw.edu.pl (P.K.)

* Correspondence: dnieciecka@chem.uw.edu.pl

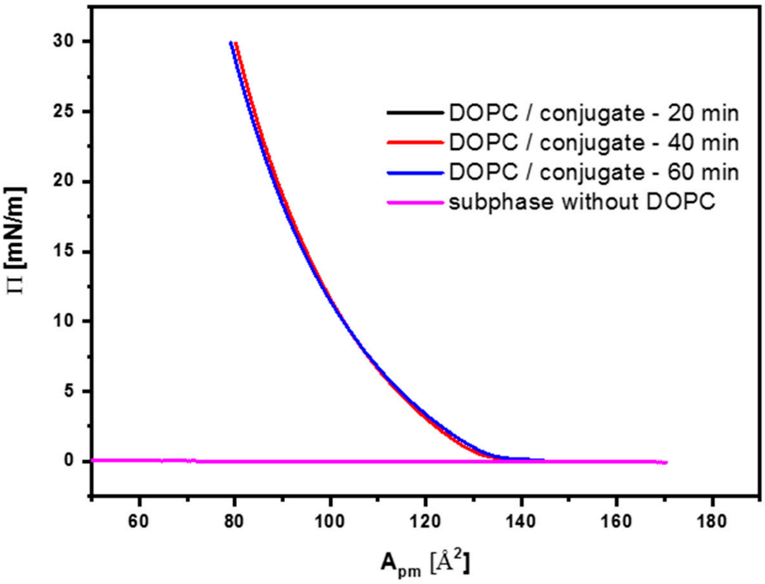

Figure S1. Langmuir isotherm for lipid DOPC (concentration of conjugate in the subphase 14 mg/L)

Compression-decompression isotherms recorded in time follow the same shape, showing that the monolayer system attained the equilibrium state after 20 min required for solvent evaporation.

GMP release studies

2 mg of conjugate was suspended in a volume (5 ml) of phosphate buffer saline (PBS) at various pH (pH 5 and pH 7) at room temperature. The resulting suspension was placed in a vial for 7 h and 100 µl were taken out of the solvent at appropriate time intervals, replaced by the same volume of fresh PBS buffer, to keep the total volume of the release medium constant. Before each sampling (100 µl), the conjugate

was sedimented on the permanent neodymium magnet. 100 µl of supernatant was diluted of water to 2 ml in cuvette.The amount of GMP released was recorded by UV spectrophotometer at 252 nm

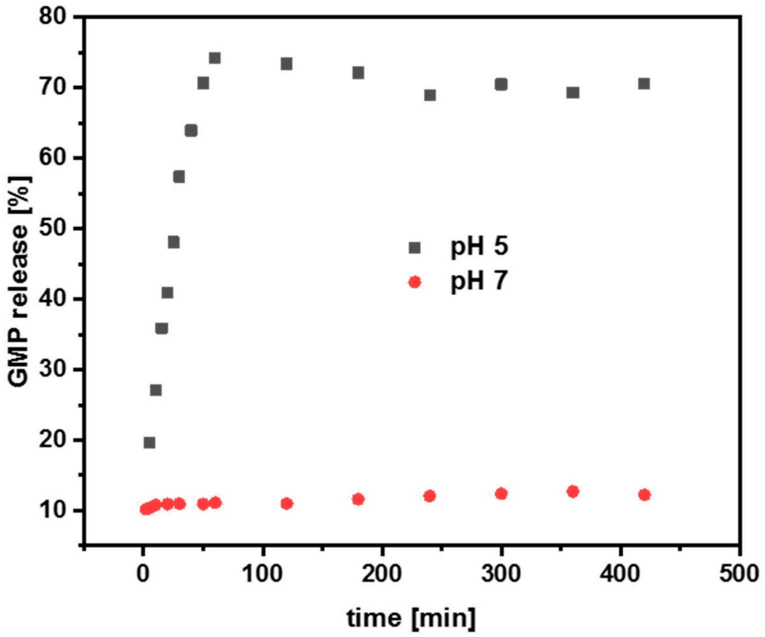

Figure S2. Release profile of GMP from conjugate at pH 5 (black squares) and pH 7 (red circles)

Cytotoxicity results

Results

Tested conjugatess show very low to none toxicity towards MDA-MB-231 cell line after 24h (114,9-91,8% metabolic activity). Toxicity increases after 48h of incubation for concentrations of 3,13 µg/mL to 100 µg/mL (73,12-66,15%). The viability decrease of untreated cells after 48 h is somehow surprising (83,6%), thus this preliminary experiment should be repeated. Viability decreases to as low as 64,91% for concentrations range 25-100 µg/mL. Therefore, for future experiments with guanosine in place of GMP, concentrations up to 5 µg/mL of nanoconjugates will be used.

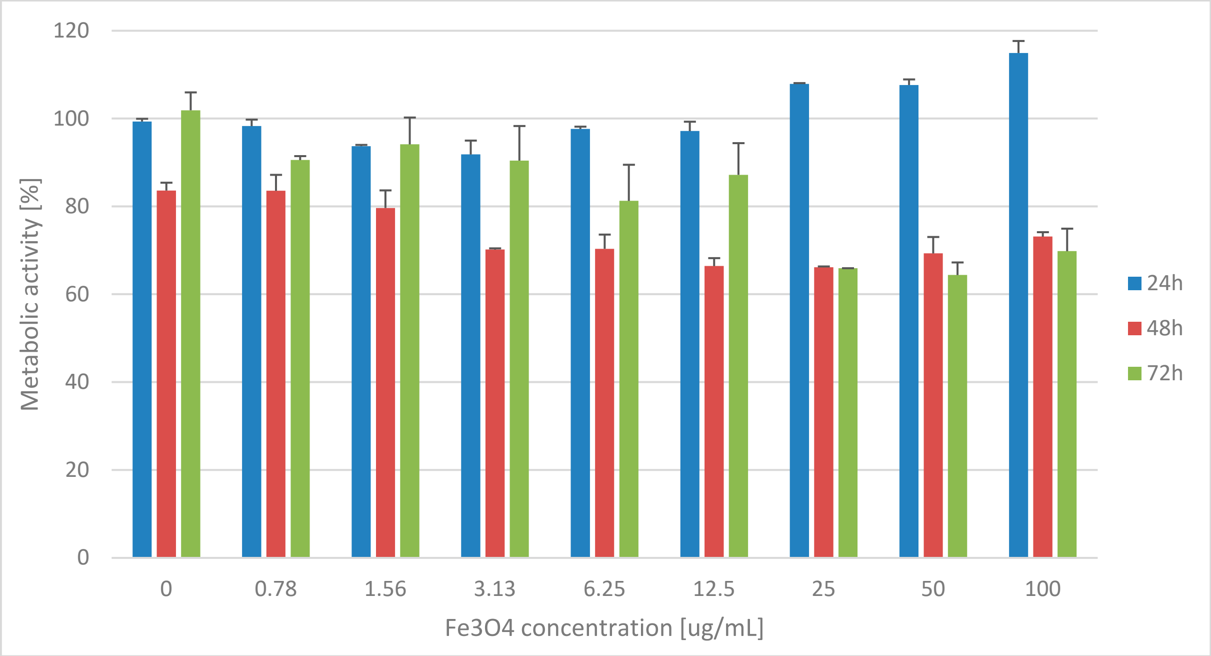

Figure S3. Percentage of metabolic avtivity on mDA-MB-231 cell line treated with IONP@CA_GMP_TB^{3+} after 24 h, 48 h and 72 h.
